# Obstructive Sleep Apnoea in Patients Treated for Head and Neck Cancer: A Systematic Review of the Literature

**DOI:** 10.3390/medicina56080399

**Published:** 2020-08-08

**Authors:** Massimo Ralli, Flaminia Campo, Diletta Angeletti, Eugenia Allegra, Antonio Minni, Antonella Polimeni, Antonio Greco, Marco de Vincentiis

**Affiliations:** 1 Department of Sense Organs, Sapienza University of Rome, 00185 Rome, Italy; flaminiacampo@gmail.com (F.C.); diletta.angeletti@uniroma1.it (D.A.); antonio.minni@uniroma1.it (A.M.); antonio.greco@uniroma1.it (A.G.); 2 Otolaryngology Department of Health Science, Magna Graecia University of Catanzaro, 88100 Catanzaro, Italy; eualle@unicz.it; 3 Department of Oral and Maxillofacial Sciences, Sapienza University of Rome, 00185 Rome, Italy; antonella.polimeni@uniroma1.it (A.P.); marco.devincentiis@uniroma1.it (M.d.V.)

**Keywords:** obstructive sleep apnoea, head and neck cancer, systematic review

## Abstract

*Background and objectives*: Obstructive sleep apnoea (OSA) is clinically defined by signs of daytime sleepiness and objective measures of disordered breathing during sleep. The literature is still controversial on the incidence and aetiology of OSA secondary to head and neck cancer treatment. The aim of this systematic review is to evaluate and discuss the prevalence of OSA in patients treated with surgery and/or chemo/radiotherapy for head and neck cancer. *Materials and methods*: Following the Preferred Reporting Items for Systematic Reviews and Meta-Analyses (PRISMA) guidelines, a systematic search was performed on May 2020 using the MEDLINE database, Scopus, and Google Scholar. The searches were conducted using combinations of the following terms: head and neck cancer, OSA, radiotherapy, chemotherapy, partial laryngectomy, laryngeal cancer, neoplasm, tumour, carcinoma, and oropharyngeal cancer. *Results*: Our results suggest that head and neck cancer patients have a higher incidence of OSA (59.78%) compared to the general population; differences may occur based on the type of treatment. *Conclusions*: Clinicians should recognise the higher prevalence of OSA in patients treated for head and neck cancer and should consider a comprehensive sleep history as part of the evaluation and management of these patients. Further research is needed to evaluate the exact prevalence, aetiology, and correct management of OSA after treatment for head and neck cancer.

## 1. Introduction

The most frequent type of sleep-disordered breathing in industrialised societies is obstructive sleep apnoea (OSA), with an incidence ranging between 3% and 7% in male and 2% and 5% in female middle-aged adults [1,2,3]. Children are also commonly affected [4,5,6]. OSA is clinically defined by signs of daytime sleepiness and objective measures of disordered breathing during sleep [4]. The main diagnostic factor for OSA is recurrent upper airway obstruction during sleep, causing repetitive apnoea episodes accompanied by oxygen desaturation and arousal from sleep [7,8,9].

OSA can provoke significant morbidity and mortality, and it has been related to severe daytime hypersomnolence, automobile accidents, and cardiovascular complications [10,11]. Quality of life can also be severely affected [12,13,14]. The aetiopathogenesis of OSA is based on the difficulty of keeping patent upper airways during sleep; causes include (1) the loss of function of the pharyngeal dilator musculature; (2) the narrowing of the pharyngeal space secondary to anatomical abnormalities; and (3) the prominence of tongue base and palate [15,16,17].

Surgery and chemo/radiotherapy for head and neck cancer may lead to several alterations of the anatomical structure and functionality of the upper airways: radiotherapy is responsible for oedema of the soft tissues, while partial laryngectomy and tongue reconstruction can change the main supporting structures of the pharynx and larynx. However, only a few studies have focused on OSA following treatment for head and neck cancer and its impact on quality of life [18], and the literature is still controversial on the incidence and aetiology of OSA in these patients.

The aim of this systematic review is to evaluate and discuss the prevalence of OSA in patients treated for head and neck cancer.

## 2. Methods

### 2.1. Search Strategy

Following the Preferred Reporting Items for Systematic Reviews and Meta-Analyses (PRISMA) guidelines, a systematic search was performed in May 2020 using the MEDLINE database, Scopus, and Google Scholar.

The searches were conducted using combinations of the following terms: head and neck cancer, OSA, radiotherapy, chemotherapy, partial laryngectomy, laryngeal cancer, neoplasm, tumour, carcinoma, and oropharyngeal cancer.

The inclusion criteria were clinical studies with abstracts available in English with patients treated for head and neck cancer, and with the data reported from an all-night-attended, comprehensive sleep study performed using a computerised polygraph.

No patients who received total laryngectomy or that were breathing with the assistance of a tracheotomy/tracheostomy were included in the review; furthermore, studies where the Apnoea-Hypopnoea Index (AHI) was not reported and studies with less than 10 patients were excluded.

Titles and abstracts were watchfully examined independently by the authors according to the inclusion and exclusion criteria, and duplicates were removed. The full text of the included studies was reviewed with extraction of following data: (1) number of patients; (2) incidence of OSA, (3) surgery treatment, (4) chemo/radiotherapy treatment, (5) AHI.

### 2.2. Study Quality

According to the standards by Wasserman et al. [19], the levels of evidence of the included articles were scored as follows: Level I: randomised controlled trials; level II: Prospective study with internal control group; level III: Retrospective study with internal control group; level IV: Case series without an internal control group; and level V: consensus or expert opinion without critical appraisal.

## 3. Results

### 3.1. Search Results

**Figure 1:**
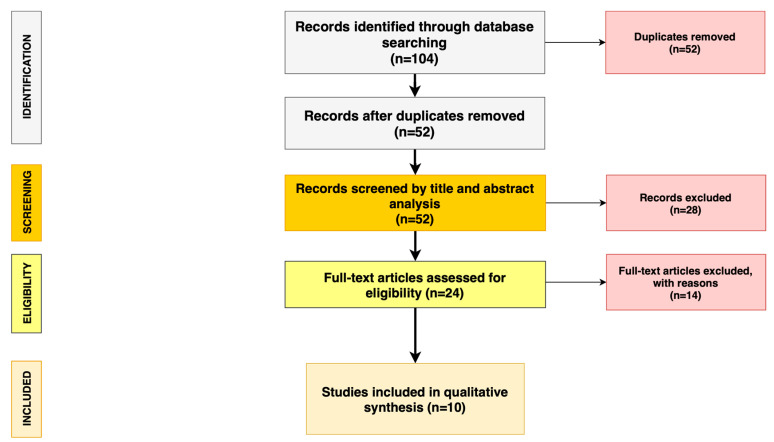
Preferred Reporting Items for Systematic Reviews and Meta-Analyses (PRISMA) diagram followed in this review. The diagram shows the information flow through the different phases of the review and illustrates the number of records that were identified and included.

The search algorithm and review results are outlined in Figure 1.

The initial search found 104 studies on the MEDLINE database, Scopus, and Google Scholar. The removal of duplicates identified 52 publications. All the 52 papers were screened in title and abstract, and 24 manuscripts were reviewed in full text.

Ten studies met the inclusion criteria (Table 1), while 14 studies did not meet the inclusion criteria and were excluded (Supplementary Material 1 Table S1). The included studies were published in peer-reviewed journals and were case series without an internal control group, with level IV evidence. No randomised controlled trial studies were identified.

### 3.2. Data Synthesis and Analysis

Given the heterogeneity among included studies, a formal meta-analysis could not be appropriately performed. The data from each study were transcribed in a tabular form. Two hundred and seventy patients treated for head and neck cancer and that performed a polysomnography were present in the 10 included studies (Table 1).

Included studies showed that OSA (AHI > 5) in head and neck patients has a spectrum of incidence that ranges from 12% to 95.8%, with a weighted average of 59.78.

One study [27] analysed patients treated only with non-surgical methods; the study included 16 patients who underwent radiation therapy for oropharyngeal or laryngeal squamous cell carcinoma. Authors diagnosed OSA ranging from mild to severe in 50% of patients (Table 2).

Two studies analysed the incidence of OSA in patients with head and neck cancer treated only with surgery [22,28]. These studies included only laryngeal function preservation surgery and found an OSA incidence near 85%. Patients undergoing open partial laryngectomy II (OPHL II) had an AHI index between 17.47 and 20.6, while patients undergoing vertical partial laryngectomy (VPL) had an AHI index ranging from 9.66 to 18.2. Both studies concluded that partial laryngectomy results in OSA by altering the anatomical structures of the larynx and pharynx [22,28] (Table 3).

Loth et al. and Qian et al. evaluated the prevalence of OSA in a population of patients with head and neck cancer, according to the treatment strategy (chemo/radiotherapy or surgery). They obtained contrasting results [11,24] (Table 4). Loth et al. [11] found that independently from the treatment strategy, head and neck cancer patients were at risk of developing OSA, with an incidence of 30% for patients treated with surgery and of 24.39% for patients treated with chemo/radiotherapy, and with a negative impact on quality of life. Contrarily, Qian et al. [24] reported that patients undergoing surgery had a higher risk of developing moderate to severe OSA in the postoperative period (73%), compared to a nonsurgical group (33.3%).

The other five studies analysed the incidence of OSA in patients with head and neck cancer treated either with chemo and/or radiotherapy and surgery. All of them concluded that patients treated for advanced head and neck cancer had a higher risk of OSA, ranging from 12% to 92.3%, and testing for OSA should be considered in these patients (Table 5) [20,21,25,26,28].

## 4. Discussion

Patients with OSA may refer loud snoring, oxygen desaturation, frequent arousals, and disruption of sleep. OSA is also being documented as an independent risk factor for stroke, hypertension, coronary heart disease, and abnormal glucose metabolism [29,30,31,32]. Even more, in the last 10 years, experimental studies have advised that OSA might supply the tumour growth and metastatisation [33]; in fact, numerous mechanisms may connect OSA with cancer incidence and prognosis such as systemic inflammation, sympathetic over-activity, angiogenesis, and immunological alterations [33]. A recent study has linked severe OSA with an amplified risk of cancer mortality in patients with stage III–IV lung cancer [34], while another research has identified the disease as a risk factor for breast cancer in women [35].

Payne et al. [36] studied the prevalence of OSA in patients with cancer of the oral cavity and oropharynx undergoing primary surgical resection. The authors found a robust connection between OSA and malignancies of the oral cavity and oropharynx. When comparing two groups (AHI < 20 and AHI > 20), there was a propensity for the group with an AHI > 20 to have increased risk of postoperative complications.

The results of our systematic review show that OSA is a relatively common disease in general population, but it is far more common in head and neck cancer patients with an incidence ranging from 12% to 95.8% and a weighted average of 59.78%. The identification and treatment of OSA may represent a main factor to improve quality of life, morbidity, and mortality. However, polysomnography is usually not executed before head and neck cancer treatment, since it is not included in routine preoperative diagnostic workup.

Over time, the aim of the management of head and neck cancer has changed to obtain the best oncological and functional outcomes with increasing attention to side effects and quality of life [37]. Patients with head and neck cancer have numerous reasons for fatigue and possibly hypersomnolence. Surgical and radiation treatment are debilitating, and recovery is often protracted [38]. Poor nutritional intake, depression, anxiety, and pain can be other causes of fatigue. OSA may worsen this condition and, if present, it should be promptly identified and treated in patients at risk [39].

The anatomic alterations in head and neck cancer patients are a significant risk factor for developing or worsening OSA. In these patients, the anatomy of the larynx and pharynx is considerably altered by the cancer mass, chemo/radiotherapy, and surgery.

Huyett et al. [27] studied irradiated laryngeal and oropharyngeal cancer patients and the spectrum of anatomic and functional changes occurring during therapy. They suggested that radiotherapy, causing oedema, may be a risk factor for OSA. Interestingly, they noticed that patients with OSA had a moderately shorter time interval between conclusion of radiation and sleep study date, advising that more immediate post-radiation changes (oedema) may make a patient more susceptible to developing OSA than the later post-radiation changes (fibrosis) [27]. Furthermore, the oedema may be worsened by hypothyroidism following radiation therapy [40]. Lastly, damage to the salivary glands can lead to a lack of saliva, reducing lubrication of the oral and oropharyngeal mucosa, thereby increasing upper airway resistance [41].

Similarly, several factors may contribute to the increased incidence of OSA in patients treated for larynx or tongue cancer with surgery. Ouyang et al. [28] studied the anatomical changes in patients with partial laryngectomy and OSA. The authors showed that after partial laryngectomy, the thyroid cartilage, which maintains open the laryngeal cavity, is replaced by soft tissue that collapses more frequently during inhalation. Furthermore, partial laryngectomy alters the main supporting structures of the hypopharynx, including the constrictor muscles of pharynx, thus causing upper airway hypotonia during sleep followed by OSA [42]. The authors concluded that the scar tissues that persist after recovery did not have the same tension of the original neuromuscular tissue, which collapsed during sleep and led to OSA [28].

Combined treatment has also been shown to increase the risk of developing OSA. Gilat et al. analysed the incidence of OSA in patients treated for tongue cancer with both surgery (partial glossectomy) and radiotherapy [25]. The authors found that the mean Epworth Sleepiness Scale (ESS) score was 8.18  ±  6.18, and 53.3% of patients had OSA (5 mild, 2 moderate, 1 severe). They concluded that the main causes of sleep disorders were the anatomic alterations after surgery. In fact, the radial forearm flaps commonly used for reconstruction of the tongue represent an enlarged soft tissue in the oral cavity, and the sensorium of the reconstructed area is rarely recovered. Moreover, OSA may be triggered by partial loss of the dilator and retractor musculature, such as the palatoglossus and geniohyoid muscles, which are responsible for precluding the tongue from collapsing backward during sleep [25].

The results of our review confirm the high prevalence of sleep-disordered breathing in patients treated for head and neck cancer [43] and suggest that an active attitude to recognise OSA in these patients may help reducing their overall symptom burden.

The use of an established sleep survey as a supplement to the clinician’s simple sleep history could help recognising patients at risk. Among the articles that we included in the present review, the most used questionnaire is ESS, followed by other screening tools such as the Berlin questionnaire (BQ), STOP-BANG questionnaire (SBQ), and the STOP questionnaire (STOP) [11,21,23,24,25,27,28]. The ESS questionnaire is an extensively used self-reported measure of daytime sleepiness that has been used with good results in several studies [11,21,23,24,25,27,28]. Contrarily, Chiu et al. reported that the SBQ questionnaire is a more accurate tool to detect mild, moderate, and severe OSA, helping to conduct patient interviews for the early diagnosis of OSA in a clinical setting [44].

Several protocols can be used for the management of OSA patients following treatment for head and neck cancer. Laser microsurgery can be used to remove excess mucosa and improve airway patency in patients with stenosis at the neo-glottis level after partial laryngectomy [21]. OSA caused by the narrowing of the retrolingual space can be treated with coblation channeling at the tongue base, continuous positive airway pressure (CPAP), and oral appliances.

Patients with head and neck cancer and OSA also may be more difficult to treat. CPAP compliance may be limited by pain and xerostomia; oral appliance therapy compliance may be reduced by trismus, xerostomia, and dental changes [20,45]. Chronic opioid consumption, common for pain management in these patients, has been revealed to worsen sleep-disordered breathing through central mechanisms [46].

### Limitations of the Study

The current study has some limitations. They include the small number of the articles that matched our inclusion criteria and incomplete data on important cofactors of interest, such as primary tumour location, body mass index, thyroid function, and the absence of pre-treatment and post-treatment analysis.

## 5. Conclusions

The present systematic review suggests that OSA may be more common in head and neck cancer patients compared to the general population. Head and neck oncology clinicians should recognise the higher prevalence of sleep-disordered breathing in these patients and consider a comprehensive evaluation of sleep disorders as part of their general management. Further research is needed to evaluate specific diagnostic protocols for OSA in cancer patients, define the correct management for OSA following treatment for head and neck cancer, and assess whether OSA treatment in these patients may improve cancer prognosis.

## Figures and Tables

**Table 1:** Studies included in our review. For each study, design and diagnostic tools are indicated. ESS: Epworth Sleepiness Scale; FOSQ-10: Functional Outcomes in Sleep Questionnaire; UW QOL: University of Washington Quality of Life survey; EORTC-QLQ-C30: European Organisation for the Research and Treatment of Cancer Quality of Life Questionnaire; EORTC H&N35: European Organisation for the Research and Treatment of Cancer head and neck.

Authors	Study Design	Diagnostic Tool(s)
Friedman et al. 2001 [20]	Prospective cohort	Polysomnography
Nesse et al. 2006 [21]	Prospective cohort	Polysomnography (Embla A10 digital recorder (Medcare, Reykjavik, Iceland), ESS, 5-item questionnaire
Israel et al. 2006 [22]	Prospective cohort	ESS, polysomnography (Meditron unit with 13 recorded channels).
Steffen et al. 2009 [23]	Prospective cohort	ESS, polysomnography (Somnocheck, Weinmann Inc., Hamburg Germany)
Qian et al. 2010 [24]	Prospective cohort	Polysomnography, ESS
Gilat et al. 2013 [25]	Prospective cohort	ESS, polysomnography in the sleep lab (Compumedics e-Series, Profusion device, version 1.01; Compumedics Ltd., Abbotsford, Victoria)
Teixeira et al. 2013 [26]	Prospective cohort	ESS, polysomnography in the sleep lab, Spirometry
Huyett et al. 2017 [27]	Prospective cohort	Polysomnography four-channel home sleep test (ResMed ApneaLink, San Diego, CA), ESS, FOSQ-10, UW QOL
Loth et al. 2017 [11]	Prospective cohort	ESS, EORTC QLQ-C30 and the EORTC H&N35, Home overnight respiratory polysomnography (Alice PDx & Sleep- ware G3 device, Respironics, Philips)
Ouyang et al. 2019 [28]	Prospective cohort	Polysomnography in the sleep lab, Flexible pharyngoscopy with Müller’s maneuver, ESS, Computed tomography, Sleep dynamic magnetic resonance imaging

**Table 2:** Incidence of OSA in patients with head and neck cancer treated with radiotherapy. n: Number; AHI: Apnoea–Hypopnoea Index; OSA: Obstructive sleep apnoea.

Study	Patients (*n*)	OSA incidence (AHI > 5)	AHI
Huyett et al. 2017 [27]	16	50%	5.3

**Table 3:** Incidence of OSA in patients with head and neck cancer treated with surgery. OPHL II: Open partial laryngectomy II; VPL: Vertical partial laryngectomy; n: number; AHI: Apnoea–Hypopnoea Index; OSA: Obstructive sleep apnoea.

Study	Patients (n)	OSA Incidence (AHI > 5)	OPHL II (*n*)	OPHL II (AHI)	VPL (*n*)	VPL (AHI)
Ouyang et al. 2019 [28]	40	82.5%	24	17.47 ± 8.73	16	9.66 ± 6.01
Israel et al. 2006 [22]	22	86.3%	11	20.6 ± 17.8	11	18.2 ± 22.2

**Table 4:** Incidence of OSA according to the treatment strategy (surgical/not surgical). RT: Radiotherapy; CT: Chemotherapy; n: number; AHI: Apnoea–Hypopnoea Index; OSA: Obstructive sleep apnoea.

Study	Patients (*n*)	OSA Incidence (AHI > 5)	Surgery ± RT/CT	OSA Incidence (Surgery)	RT/CT	OSA Incidence RT/CT	Outcome
Loth et al. 2017 [11]	51	25.49%	10	30%	41	24.39%	No difference between RT/CT and surgery
Qian et al. 2010 [24]	24	59.78%	15	AHI >15 in 73%	9	AHI >15 in 33.3%	OSA more frequent in patient treated with surgery

**Table 5:** Incidence of OSA in patients with head and neck cancer treated either with radiotherapy ad surgery. n: Number; AHI: Apnoea–Hypopnoea Index; OSA: Obstructive sleep apnoea; NA: Not available.

Study	Patients (n)	OSA Incidence	AHI
Friedman et al. 2001 [20]	24	AHI > 5 in 91.7%	49.86
Nesse et al. 2006 [21]	33	AHI > 5 in 12%	NA
Steffen et al. 2009 [23]	31	AHI > 20 in 19%	NA
Teixeira et al. 2013 [26]	14	AHI > 5 in 92.3%	24
Gilat et al. 2013 [25]	15	AHI > 5 in 53.30%	NA

## Supplementary Materials

The following are available online at https://www.mdpi.com/1010-660X/56/8/399/s1, Table S1: List of articles excluded from our review because they did not match the inclusion criteria.
