# Supplementary material for: Obstructive Sleep Apnoea in Patients Treated for Head and Neck Cancer: A Systematic Review of the Literature
Source: Medicina (Kaunas). 2020 Aug 8;56(8):399. doi: 10.3390/medicina56080399 (PMC7466207; doi:10.3390/medicina56080399)
Supplement: Supplementary file 1 [file medicina-56-00399-s001.pdf]

Supplementary Material S1: List of articles excluded from our review because they did not match the inclusion criteria.

Authors	Reasons for Exclusion
Saesen et al. 2020 [1]	Presence of OSA was estimated using only questionnaires
Garas et al. 2018 [2]	Incomplete data on head and neck cancer and OSA
Chung et al. 2018 [3]	Patients underwent transsphenoidal pituitary surgery
Kirca et al. 2017 [4]	Presence of OSA was estimated using only questionnaires
Unal et al. 2016 [5]	No evaluation of OSA
Ninu et al. 2016 [6]	Presence of sleep disorders was estimated using only questionnaires
Zhou et al. 2015 [7]	Review article
Faiz et al. 2014 [8]	Type of treatment is not specified
Richmond et al. 2014 [9]	Incomplete data on head and neck cancer and OSA
Dedhia et al. 2014 [10]	Review article
Rosenthal et al. 2014[11]	Presence of sleep disorders was estimated using only questionnaires
Payne et al. 2005[12]	Patients were approached to undergo overnight polysomnography exclusively before surgery
Rada et al. 2004[13]	Review article
Koliha et al. 2003[14]	Review article

1. Saesen, K.; van der Veen, J.; Buyse, B.; Nuyts, S. Obstructive sleep apnea in head and neck cancer survivors. *Support. Care Cancer* **2020**, doi:10.1007/s00520-020-05428-7.
2. Garas, G.; Arora, A. Robotic Head and Neck Surgery: History, Technical Evolution and the Future. *ORL J. Otorhinolaryngol. Relat. Spec.* **2018**, *80*, 117–124, doi:10.1159/000489464.
3. Chung, S.Y.; Sylvester, M.J.; Patel, V.R.; Zaki, M.; Baredes, S.; Liu, J.K.; Eloy, J.A. Impact of obstructive sleep apnea in transsphenoidal pituitary surgery: An analysis of inpatient data. *Laryngoscope* **2018**, *128*, 1027–1032, doi:10.1002/lary.26731.
4. Kirca, K.; Kutluturkan, S. Symptoms of patients with head and neck cancers undergoing radiotherapy. *Eur. J. Cancer Care (Engl.)* **2017**, *26*, doi:10.1111/ecc.12584.
5. Unal, D.; Orhan, O.; Ozsoy, S.D.; Besirli, A.; Eroglu, C.; Kaplan, B. Effect of radiotherapy on psychiatric disorder in patients with head and neck cancer. *Indian J. Cancer* **2016**, *53*, 162–165, doi:10.4103/0019-509X.180816.
6. Ninu, M.B.; Miccinesi, G.; Bulli, F.; De Massimi, A.; Muraca, M.G.; Franchi, G.; Squadrelli Saraceno, M. Psychological distress and health-related quality of life among head and neck cancer patients during the first year after treatment. *Tumori* **2016**, *102*, 96–102, doi:10.5301/tj.5000448.
7. Zhou, J.; Jolly, S. Obstructive sleep apnea and fatigue in head and neck cancer patients. *Am. J. Clin. Oncol.* **2015**, *38*, 411–414, doi:10.1097/01.coc.0000436086.61460.cb.
8. Faiz, S.A.; Balachandran, D.; Hessel, A.C.; Lei, X.; Beadle, B.M.; William, W.N., Jr.; Bashoura, L. Sleep-related breathing disorders in patients with tumors in the head and neck region. *Oncologist* **2014**, *19*, 1200–1206, doi:10.1634/theoncologist.2014-0176.
9. Richmon, J.D.; Feng, A.L.; Yang, W.; Starmer, H.; Quon, H.; Gourin, C.G. Feasibility of rapid discharge after transoral robotic surgery of the oropharynx. *Laryngoscope* **2014**, *124*, 2518–2525, doi:10.1002/lary.24748.
10. Dedhia, R.C.; Rosen, C.A.; Soose, R.J. What is the role of the larynx in adult obstructive sleep apnea? *Laryngoscope* **2014**, *124*, 1029–1034, doi:10.1002/lary.24494.
11. Rosenthal, D.I.; Mendoza, T.R.; Fuller, C.D.; Hutcheson, K.A.; Wang, X.S.; Hanna, E.Y.; Lu, C.; Garden, A.S.; Morrison, W.H.; Cleeland, C.S.; et al. Patterns of symptom burden during radiotherapy or concurrent chemoradiotherapy for head and neck cancer: A prospective analysis using the University of Texas MD Anderson Cancer Center Symptom Inventory-Head and Neck Module. *Cancer* **2014**, *120*, 1975–1984, doi:10.1002/cncr.28672.

12. Payne, R.J.; Hier, M.P.; Kost, K.M.; Black, M.J.; Zeitouni, A.G.; Frenkiel, S.; Naor, N.; Kimoff, R.J. High prevalence of obstructive sleep apnea among patients with head and neck cancer. *J. Otolaryngol.* **2005**, *34*, 304–311, doi:10.2310/7070.2005.34502.
13. Rada, R. Obstructive sleep apnea and head and neck neoplasms. *Otolaryngol. Head Neck Surg.* **2005**, *132*, 794–799, doi:10.1016/j.otohns.2004.12.002.
14. Koliha, C.A. Obstructive sleep apnea in head and neck cancer patients post treatment ... something to consider? *ORL Head Neck Nurs.* **2003**, *21*, 10–14.
